# Impact of chronic outward force on arterial responses of proximal and distal of long superficial femoral artery stent

**DOI:** 10.1186/s12872-021-02141-z

**Published:** 2021-06-30

**Authors:** Hu Li, Seung-Woon Rha, Byoung Geol Choi, Se Yeon Choi, Sang Ki Moon, Won Young Jang, Woohyeun Kim, Ji Hun Ahn, Sang-Ho Park, Woong Gil Choi, Rui Feng Yang, Wen Wei Bai, Cheol Ung Choi, Yang gi Ryu, Man Jong Baek, Dong Joo Oh

**Affiliations:** 1 Department of Medicine, The Second Affiliated Hospital of Kunming Medical University, Kunming, Yunnan China; 2 Cardiovascular Center, Korea University Guro Hospital, 148, Gurodong-ro, Guro-gu, Seoul, 08308 Republic of Korea; 3 Department of Cardiology, Soon Chun Hyang University Gumi Hospital, Gumi-si, Republic of Korea; 4 Department of Cardiology, Soonchunhyang University Cheonan Hospital Korea, Cheonan-Ii, Republic of Korea; 5 Department of Internal Medicine, School of Medicine, Konkuk University, Chungju, Republic of Korea

**Keywords:** Chronic outward force, Superficial femoral artery, Self-expanding nitinol stents, Stent oversizing, Histomorphometry

## Abstract

**Background:**

Self-expanding nitinol stent (SENS) implantation is commonly oversized in the superficial femoral artery (SFA), and leads to chronic outward force (COF) and in-stent restenosis (ISR). This study aimed to investigate the impact of COF of oversizing SENS on ISR of SFA.

**Methods:**

In patients with implanted SENS in SFA, intimal hyperplasia especially between proximal segment and distal segment was evaluated by quantitative angiography, and the impact of COF on mid-term angiographic outcomes was investigated. In addition, porcine model with implanted SENS was used to evaluate the impact of COF on angiographic and histopathologic outcomes at 1 month. Excised stented arteries were evaluated by histopathologic analysis.

**Results:**

We analyzed 65 SENS in 61 patients with follow-up angiography at 6 months to 1 year. The baseline diameter was 6.8 ± 0.71 mm and length were 97.0 ± 33.8 mm for the SENS. The ratio of the diameter of the stent to the reference vessel was 1.3 ± 0.24 at the proximal portion and 1.53 ± 0.27 at the distal portion (*P* < 0.001). In the long SFA stent, stent-to-vessel ratio was significantly higher in the distal stent than in the proximal stent (1.3 ± 0.2 vs. 1.55 ± 0.25, *P* = 0.001). ISR incidence was higher at the distal stent (37.3% vs 52.6%, *P* = 0.029). All 11 pigs survived for 4 weeks after SENS implantation. The vessel diameter was 4.04 ± 0.40 mm (control group) vs 4.45 ± 0.63 mm (oversized group), and the implanted stent diameter was 5.27 ± 0.46 mm vs. 7.18 ± 0.4 mm (*P* = 0.001). The stent-to-vessel diameter ratio was 1.31 ± 0.12 versus 1.63 ± 0.20 (*P* < 0.001). After 4 weeks, restenosis % was 29.5 ± 12.9% versus 46.8 ± 21.5% (*P* = 0.016). The neointimal area was 5.37 ± 1.15 mm^2^ vs. 8.53 ± 5.18 mm^2^ (*P* = 0.05). The restenosis % was 39.34 ± 8.53% versus 63.97 ± 17.1% (*P* = 0.001).

**Conclusions:**

COF is an important cause of restenosis in the distal portion of the SFA stent. Optimal sizing of the SFA stent is important to reduce the incidence of restenosis. Therefore, COF was an important factor of restenosis following distal SFA stenting.

## Introduction

Balloon angioplasty and stent implantation are popular in patients with peripheral artery disease (PAD) [1–3]. The self-expanding nitinol stent (SENS) for superficial femoral artery (SFA) lesions is morphologically and clinically superior to balloon angioplasty [2, 4, 5]. However, stent size influences clinical outcomes [6, 7]. Implanted SENS is commonly oversized in the SFA, especially at the distal portion due to tapered diameter of SFA for long SENS [8–10]. SFA stents are under dynamic stress to external forces including bending, twisting, torsion, elongation, foreshortening and external compression [11, 12]. The mechanical stresses to the SFA are associated with an increased risk of stent fracture and subsequent significant restenosis [11, 13]. The chronic outward force (COF) exerted by a SENS is another important factor to cause restenosis, and several investigations evaluated the effect of SENS oversizing on the risk of in-stent restenosis (ISR) [6, 14–16]. COF could produce continuous mechanical stimulation of the arterial wall, which can increase neointimal hyperplasia and lead to significant ISR [17].

This study aimed to evaluate the impact of SENS oversizing on ISR of SFA. We examined clinical and animal experiments data to determine the impact of COF on the arterial wall, and investigated the impact of SENS oversizing with respect to continued expansion following stenting and the mid-term histological impact of oversized stent on SFA.

## Methods

### Study design

**Fig. 1:**
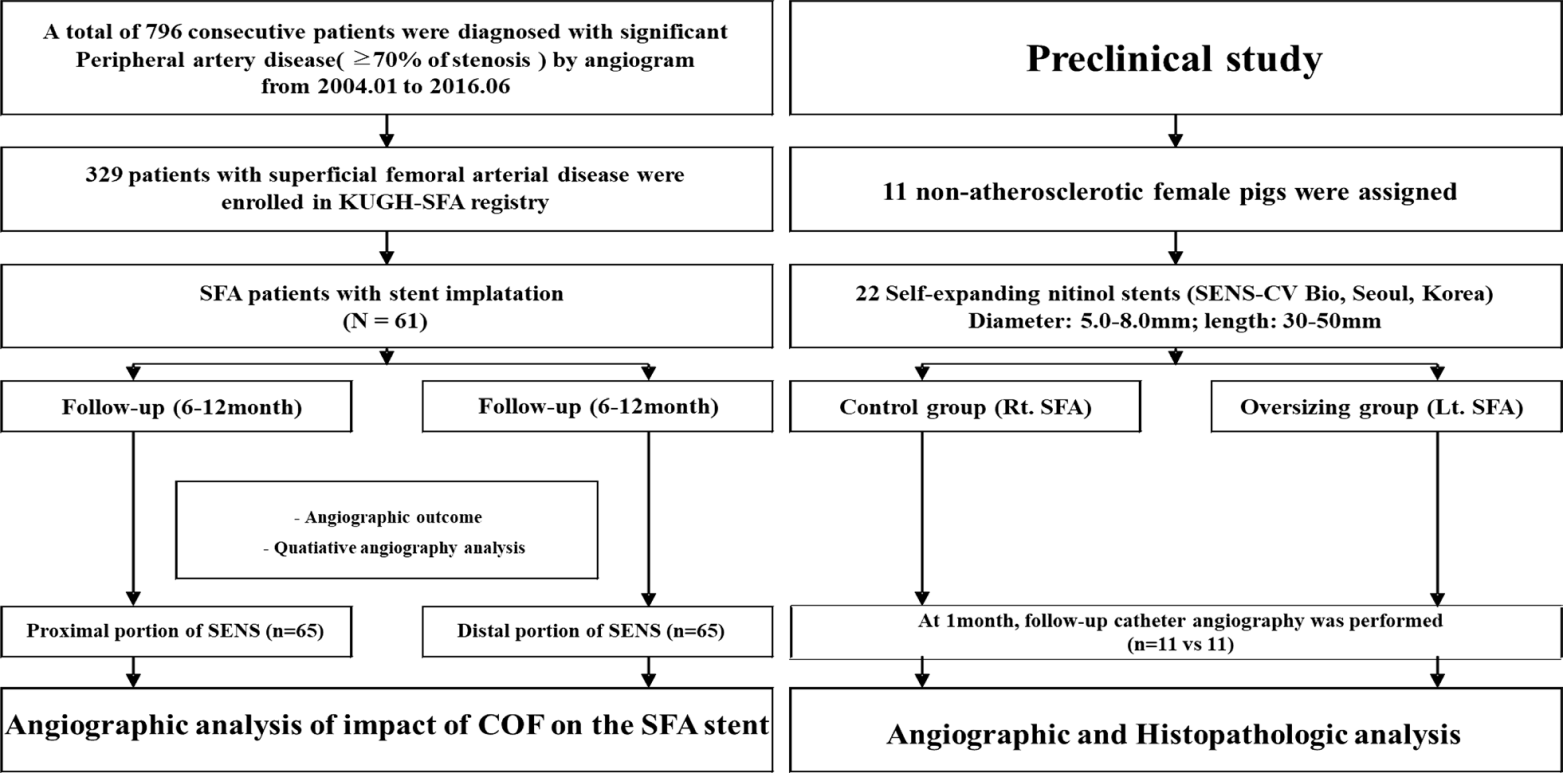
Flow chart of clinical and preclinical study

The clinical evaluation focused on intimal hyperplasia especially between the proximal and distal segments by implanted SENS in SFA. Quantitative angiography (QA) was used to determine the ISR rate following mid-term catheter angiography. The impact of COF on mid-term angiographic and clinical outcomes was investigated (Fig. 1).

Preclinical data were obtained from a porcine model following SENS (CV Bio, Seoul, Korea) implantation. The model was used to evaluate the impact of COF on angiographic outcomes and histopathologic outcomes at 1 month. Control pigs received an adequately sized SENS, whereas the COF group received an over-sized stent 1.5 to 1.8 times larger than the control size. At 4 weeks, catheter angiography and histopathologic evaluation was performed (Fig. 1).

#### Patients

The effect of COF was explored by a retrospective review of the medical records of 329 patients who underwent SENS implantation for SFA from January 2004 to June 2016. The data were obtained from the percutaneous transluminal angioplasty (PTA) registry of Korea University Guro Hospital (KUGH) (Seoul, South Korea). PTA registry is a single-center, prospective, all-comer registry designed to reflect “real world” practice since 2004. Patients had signs of a moderate intermittent claudication (Rutherford stage 2), severe intermittent claudication (Rutherford stage 3) or ischemic pain (Rutherford stage 4), or significant atherosclerotic effect in one or both femoral arteries (stenosis diameter > 70%).

Data were collected by a trained study-coordinator, following a standardized case report form. The participants or their legal guardians provided written consent to participate in the study. The study protocol was approved by Medical Review Board of KUGH.

##### Percutaneous intervention

Aspirin and clopidogrel were used in all patients and 70 U/kg heparin were given after the start of the procedure. After local anesthesia with Lidocain 2% 5F or 6F sheath was delivered through the femoral artery by the Seldinger approach. If balloon angioplasty or stenting proved technically feasible by ipsilateral antegrade approach, an antegrade puncture was preferred. Otherwise, a contralateral femoral approach or retrograde puncture from the distal SAF or proximal tibial access for bi-directional approach was used.

A 5-Fr Omni internal mammary catheter, or Judkin's right catheter was inserted into the aortic-iliac artery branch when contralateral approach was needed. The introducing soft Terumo wire was inserted into the lesion, and then the guiding sheath was placed for SFA treatment. Most frequently used guiding sheaths were 6F Ansel Checkflo (Cook, USA), 6-8F Balkin sheath (Cook, Bloomington, USA) or 8F Contralateral-1 guiding sheath (Cordis, USA).

After the successful wire crossing by 018 or 035 long wires, conventional balloon angioplasty was performed. The balloon dilatation duration was extended for at least 120 s with adequately sized balloon (4.0–7.0 mm diameter with longer balloon). Once the balloon response was not favorable, including flow limiting intimal dissection, significant residual stenosis or thrombus formation, SENS was implanted. Angiographic success was considered to be the case when residual stenosis was < 30% after the procedure and blood flow was well maintained through and distal to the target lesion.

##### Clinical and angiographic follow-up

Patients with successful intervention were clinically followed at 30 days and 3 months respectively to monitor the diabetic foot recovery or the severity of symptoms. Patients were strongly recommended for mid-term imaging follow-up by invasive or CT angiography at 6 to 12 months. QA was used to assess the impact of COF on ISR in proximal and distal SFA stents.

##### Pig COF model and procedures

Animal experiments were conducted at the K-BIO Osong Animal Research Center and the protocols were approved by Animal Care and Use Review Board (No. KBIO-IACUC-2016–005) according to the guidelines by the National Institute of Health. Eleven non-atherosclerotic female pigs (weight 25–35 kg) were assigned. Twenty self-expanding nitinol stents (SENS-CV Bio, Seoul, Korea) 5.0–8.0 mm in diameter and 30–50 mm in length were used.

Aspirin (100 mg/day) and clopidogrel (75 mg/day) were given 3 days before the procedure. On the day of the procedure, after general anesthesia with isoflurane followed by respiratory anesthesia using an animal ventilator, the right carotid artery was incised, the carotid artery was exposed, a 6-F sheath was inserted, and 200 IU/kg of heparin was injected. The 6-F guiding catheter was inserted into the SFA and baseline SFA angiography was performed. SFA in the control group was treated using SENS of the appropriate size. SFA in the COF group was treated by an oversized stent. After the procedure, the carotid artery was ligated, the pigs were transferred to the breeding farm and observed for 4 weeks. During this period, aspirin and clopidogrel were administered continuously.

The effect of COF was analyzed by angiography and histopathologic results. After 4 weeks, catheter angiography was performed through the opposite carotid artery, and QA using CAAS software was used for the evaluation of stent patency, fracture, and ISR degree. To be euthanized, pigs are given an intravenous carbon dioxide/oxygen gas mixture after general anesthesia with isoflurane and excised stented arteries were fixed in 10% formalin for 24 h for histopathologic analysis of inflammation, necrosis and intimal hyperplasia.

##### Histomorphometry

The tissues were embedded in paraffin and cut into fine section (50–100 μm) using a low-speed diamond wafer mounted on an isoment saw (Buehler Ltd., Lake Bluff, IL, USA). After hematoxylin and eosin (H&E) staining, the sections were analyzed by calibrated microscope digital video imaging system and a VISUS 2000 computerized visual image analysis system. The external elastic lamina (EEL) area, internal elastic lamina (IEL) area and the lumen area of the central, proximal and distal part of the stent from pig SFA were examined. The area stenosis of the stent vessels was conventionally calculated as: 100 × (1-lm area/IEL area).

The injury score of the vessel wall by the stent and inflammation score were evaluated by Schwartz method [18]. The injury score of the vessel wall was scored as 0 for no endothelial cell damage, 1 for the break in the internal elastic membrane, 2 for the media membrane injury, and 3 for the damage of the external elastic membrane to the adventitia. The inflammation score was scored as 0 for inflammatory cells did not infiltrate around the strut, 1 for the cells did not surround the strut, 2 for the cells did not surround the strut and 3 for tight densification.

##### Statistical analysis

All statistical analyses were performed using SPSS for Windows version 20.0 (SPSS Inc., Chicago, USA). The normality was verified via one-sample Kolmogorov–Smirnov test. Continuous variables are expressed as mean ± standard deviation (SD), categorical data were reported as frequencies and percentages. Unpaired Student's t-test were used to compare continuous variables while Chi-square test was used to compare categorical variables. Correlation between the stent/vessel ratio and the stenosis results was determined using linear regression analysis. The receiver operating characteristic (ROC) analysis was performed to get area under the curve (AUC). *P* < 0.05 was considered statistically significant.

## Results

### Clinical results

Sixty-five SENS of SFA in 61 patients with follow-up angiography at 6 months to 1 year were analyzed. The mean follow-up duration was 302 days. All PTA procedures and follow-up angiography were successful. In the most severe lesions, the minimal luminal diameter (MLD) was 0.85 ± 1.32 at the proximal lesion site mm and 0.91 ± 1.15 mm at distal lesion site (*P* = 0.619). The mean stent diameter and length was 6.8 ± 0.71 mm and 97.0 ± 33.8 mm, respectively. The ratio of the diameter of the stent to the reference vessel was 1.3 ± 0.24 at the proximal portion and 1.53 ± 0.27 at the distal portion (*P* < 0.01) (Table 1). QA of the follow-up angiograms showed a trend toward higher incidence of ISR in the distal portion of the stent. However, the mean percent restenosis was significantly higher and mean MLD was lower in the distal portion than in the proximal portion (Table 2). Subgroup analysis according to the stent length showed that for stents longer than 100 mm, the stent-to-vessel ratio and the incidence of ISR was significantly higher in the distal site than in the proximal site. The mean percent restenosis was also significantly higher in the distal site (Table 3). In contrast, for the stents shorter than 100 mm, there were no significant difference in the incidence of ISR and mean MLD between the two groups, except a trend toward higher mean percent stenosis (Table 4).

**Table 1:** Baseline angiographic characteristics

Variables (N)	Proximal of stent (n = 65)	Distal of stent (n = 65)	*P* value
RVD pre/mm	5.37 ± 1.40	4.60 ± 1.04	0.010
MLD pre/mm	0.85 ± 1.32	0.91 ± 1.15	0.619
AD pre/mm	5.40 ± 1.12	4.56 ± 0.89	0.010
Stent diameter/mm	6.80 ± 0.71	6.80 ± 0.71	1.000
Stent length/mm	97.0 ± 33.8	97.0 ± 33.8	1.000
S/A ratio	1.30 ± 0.24	1.53 ± 0.27	0.001
RVD post/mm	5.50 ± 1.26	4.91 ± 1.19	0.007
MLD post/mm	4.62 ± 1.22	4.53 ± 1.32	0.680
Stent diameter post/mm	5.17 ± 1.25	4.88 ± 1.23	0.186

**Table 2:** Mid-term angiographic outcomes regardless of stent length

Variables	Proximal of stent (n = 65)	Distal of stent (n = 65)	*P* value
ISR (%)	19 (29.2%)	29 (44.6%)	0.070
RVD FU/mm	5.29 ± 1.58	4.65 ± 1.19	0.023
MLD FU/mm	3.37 ± 1.69	2.74 ± 1.77	0.046
Stent diameter FU/mm	6.52 ± 1.52	6.10 ± 1.51	0.112
S/A ratio	1.30 ± 0.24	1.53 ± 0.27	0.001
DS% FU	36.9 ± 23.5	49.5 ± 27.6	0.006
FU duration/days	302.2 ± 12.21	302.2 ± 12.21	1.000

*ISR* in stent restenosis, *RVD* reference vessel diameter, *MLD* minimal luminal diameter, *AD* artery diameter, *S/A ratio* stent/artery ratio, *DS* diameter stenosis

**Table 3:** Mid-term angiographic outcomes in patients with longer stent (> 100 mm)

Variables	Proximal of stent (n = 23)	Distal of stent (n = 23)	*P* value
ISR (%)	5 (21.7%)	12 (52.2%)	0.032
Stent diameter/mm	6.76 ± 0.68	6.76 ± 0.68	1.000
Stent length/mm	121.35 ± 20.70	121.35 ± 20.70	1.000
RVD FU/mm	5.14 ± 1.39	4.63 ± 1.24	0.097
MLD FU/mm	3.29 ± 1.81	2.60 ± 2.08	0.110
Stent diameter FU/mm	6.34 ± 1.46	5.89 ± 1.53	0.208
S/A ratio	1.30 ± 0.20	1.55 ± 0.25	0.001
DS% FU	37.35 ± 28.82	52.61 ± 32.56	0.029
FU duration/days	302.2 ± 12.21	302.2 ± 12.21	1.000

*ISR* in stent restenosis, *RVD* reference vessel diameter, *MLD* minimal luminal diameter, *AD* artery diameter, *S/A ratio* stent/artery ratio, *DS* diameter stenosis

**Table 4:** Mid-term angiographic outcomes in patients with shorter stent (≤ 100 mm)

Variables	Proximal of stent (n = 42)	Distal of stent (n = 42)	*P* value
ISR (%)	14 (33.3%)	18 (42.9%)	0.369
Stent diameter/mm	6.85 ± 0.75	6.85 ± 0.75	1.000
Stent length/mm	65.0 ± 15.0	65.0 ± 15.0	1.000
RVD FU/mm	5.50 ± 1.80	4.68 ± 1.15	0.117
MLD FU/mm	3.48 ± 1.54	2.92 ± 1.28	0.176
Stent diameter FU/mm	6.34 ± 1.46	5.89 ± 1.53	0.326
S/A ratio	1.29 ± 0.28	1.50 ± 0.29	0.110
DS% FU	36.5 ± 20.0	45.5 ± 19.1	0.095
FU duration/days	302.2 ± 12.21	302.2 ± 12.21	1.000

*ISR* in stent restenosis, *RVD* reference vessel diameter, *MLD* minimal luminal diameter, *AD* artery diameter, *S/A ratio* stent/artery ratio, *DS* diameter stenosis

### Animal study results

**Fig. 2:**
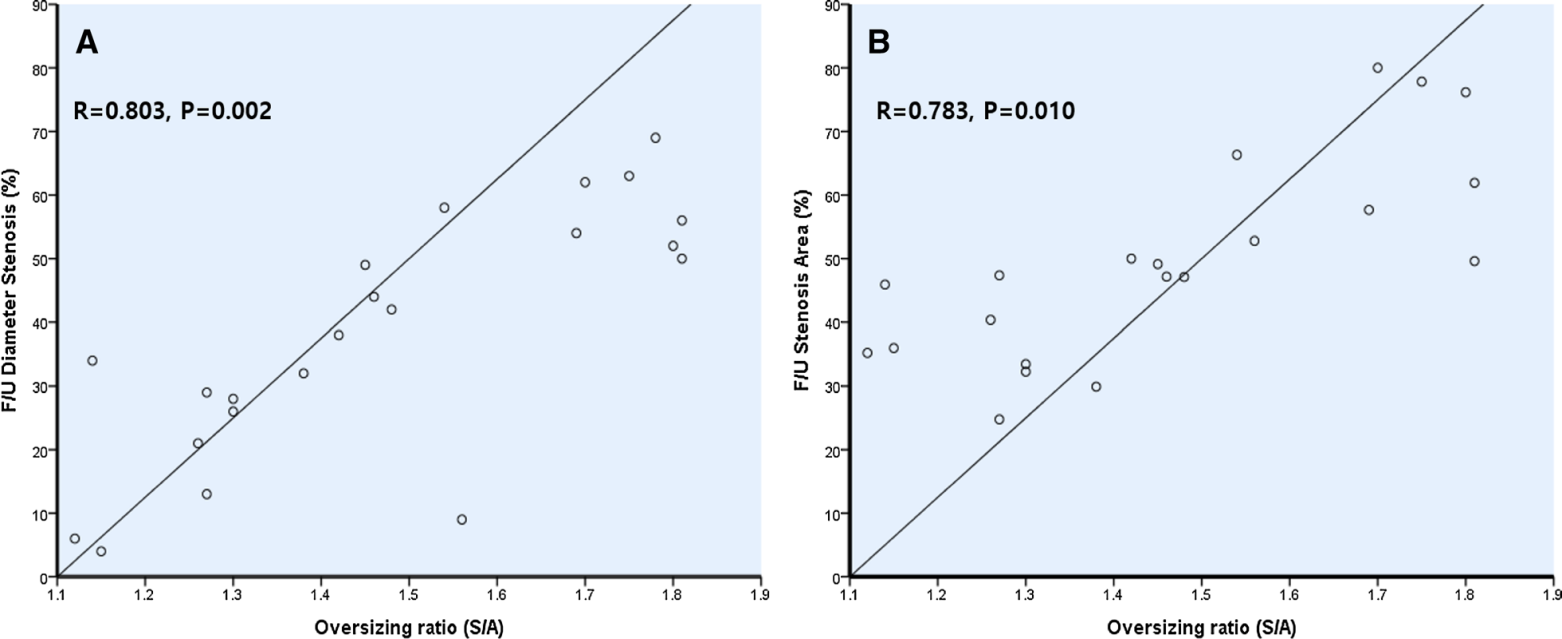
The correlation of stenosis area and the artery-to-stent oversized ratio. **a** linear correlation of oversizing ratio with in stent restenosis by QA image at 1 month; **b** linear correlation of oversizing ratio with in stent restenosis by histomorphmetry at 1 month

All 11 animals survived for 4 weeks without acute limb ischemia due to stent thrombosis. After the procedure, quantitative and morphological analyses were performed (Fig. 1). Before stenting, the vessel diameter was 4.04 ± 0.40 in the control group and 4.45 ± 0.63 in the oversized group (*P* = 0.12). The mean stent diameter implanted in the control group was 5.27 ± 0.46 mm and 7.18 ± 0.4 mm in the oversized group (*P* = 0.001). The stent-to-vessel diameter ratio was 1.31 ± 0.12 versus 1.63 ± 0.20 in control and oversized groups, respectively (*P* < 0.001) (Table 5). After 4 weeks, the incidence of ISR was higher, MLD was smaller, and the mean percentage of restenosis was higher in oversized group (Table 6). The diagnostic criteria for stent/artery ratios were 1.51 times of the standard for diagnosing the stenosis using ROC analysis (sensitivity of 0.791 and specificity of 0.929). Correlation between oversizing ratio was statistically significant (R = 0.803, *P* = 0.002). In the histopathologic examination, the incidence of ISR was higher, lumen area was smaller, neointimal and stenosis area were larger in oversized group. There was a trend toward higher inflammation score in oversized group (Table 7). In addition, the correlation of stenosis area and the artery-to-stent oversized ratio was significant (R = 0.783, *P* = 0.010) (Fig. 2).

**Table 5:** Baseline procedural characteristics in porcine model

Variables (N)	Control group (n = 11)	Oversizing group (n = 11)	*P* value
Nominal stent diameter/mm	5.27 ± 0.46	7.18 ± 0.40	0.001
Stent length/mm	30.0 ± 0.00	33.0 ± 8.09	0.476
Artery diameter/mm	4.04 ± 0.39	4.45 ± 0.63	0.120
RVD baseline/mm	4.11 ± 0.33	4.04 ± 0.47	0.936
MLD baseline/mm	4.00 ± 0.40	3.94 ± 0.51	0.573
Oversizing ratio	1.31 ± 0.12	1.63 ± 0.20	0.001

*RVD* reference vessel diameter, *MLD* minimal luminal diameter

**Table 6:** Angiographic outcomes at 1 month after stent implantation

Variables	Control group (n = 11)	Oversizing group (n = 11)	*P* value
ISR (%)	0 (0%)	8 (72.7%)	0.001
Proximal diameter FU/mm	4.45 ± 0.52	4.52 ± 0.66	0.617
Distal diameter FU/mm	3.81 ± 0.19	3.86 ± 0.68	0.640
RVD FU/mm	4.06 ± 0.24	4.17 ± 0.59	0.151
MLD FU/mm	3.16 ± 0.56	2.27 ± 0.83	0.007
Stent diameter FU/mm	4.60 ± 0.57	4.86 ± 0.69	0.340
Late loss/mm	0.83 ± 0.59	1.53 ± 1.04	0.105
DS% FU	29.5 ± 12.9	46.8 ± 21.5	0.016
FU duration /days	30.5 ± 3.50	30.5 ± 3.50	1.000

*ISR* in stent restenosis, *FU* follow up, *Stent diameter* during 1 month’s expansion of stent implantation, *RVD* reference vessel diameter, *MLD* minimal luminal diameter, *DS* diameter stenosis

**Table 7:** Comparisons of histomorphometric measurements at 1 month after stent implantation

Variables	Control group (n = 11)	Oversizing group (n = 11)	*P* value
ISR (%)	0 (0%)	8 (72.7%)	0.001
IEL/mm^2^	13.82 ± 2.33	13.09 ± 4.71	0.270
Lumen area/mm^2^	8.45 ± 2.16	4.56 ± 2.81	0.001
Neointimal area/mm^2^	5.37 ± 1.15	8.53 ± 5.18	0.050
Area stenosis/%	39.34 ± 8.53	63.97 ± 17.1	0.001
Injury score	1.0 ± 0.14	1.2 ± 0.27	0.456
Inflammation score	1.1 ± 0.12	1.4 ± 0.39	0.061

*ISR* in stent restenosis, *IEL* internal elastic lamina

## Discussion

The major findings of the impact of COF on arterial responses of SFA stent are summarized as follows. First, clinical study showed that for long SFA stent, the stent-to-artery ratio was significantly higher in the distal stent than in the proximal stent. The incidence of ISR and mean percentage of restenosis was significantly higher at the distal site, particularly in the implanted stent longer than 100 mm. Second, in animal models, the incidence of ISR was higher, lumen area was smaller, and neointimal and stenosis area were larger and there was a trend toward higher inflammation score in the oversized group. Third, the stent to artery ratio was 1.51 times of the standard for diagnosing stenosis using ROC analysis. These results support the idea that stent oversizing significantly increases the intramural stress to stimulate smooth muscle in the vessel, leading to inflammatory response and pathologic stress in the artery wall that cause irreversible damage [19].

Several clinical trials demonstrated the benefit of SENS implantation over angioplasty for stenosis or occlusion of the SFA [2, 4, 20]. However, SFA poses unique challenge for endovascular stenting. SFA is a long muscular artery that is fixed between the hip and the knee. Complex motions of the hip joint can impose complex external mechanical stresses on the SFA, including flexion, compression and torsion [11]. SFA is further exposed to longitudinal and lateral compressional stress as the artery dives through the Hunter canal between the muscle bodies of the anterior and medial compartments of the thigh [12]. Currently, a stent diameter of 1–2 mm oversizing is recommended in SFA stent implantation [8, 21]. For a self-expanding non-tapered nitinol stent, it will be implanted in accordance with the proximal diameter of the reference vessel, since the stent is self-tapered at the distal site after stenting. Therefore, the distal portion of the long SENS will exert stronger COF to distal SFA, leading to higher incidence of intimal hyperplasia and subsequent restenosis.

Previous studies reported that average reference vessel diameter of SFA is 5 mm, and most commercially available stent has a diameter of 7 mm [8–10, 22, 23]. However, SFA consists of tapered vessels. Therefore, in this procedure, a larger diameter stent will be implanted into the smaller SFA distal portion. The different diameters of the proximal and distal portions of stent impose a wall stress on the arterial wall. Vessel wall injury is an important factor during stent implantation [15]. The main reason for restenosis is adverse remodeling caused by endothelial injury, inflammation of atherosclerosis plaque, hemodynamic factors, and mechanical stress from a permanent stent [24].

The incidence of ISR depends on the type, design and length of the implanted stent. Innovative stent design may decrease ISR and improve clinical outcomes. The unique design of the Supera Peripheral Stent (Abbott Vascular, Abbott Park, IL) aimed to overcome issues related to areas of high flexion and minimal COF [9]. Recently, the Supera Interwoven Nitinol Stent Outcomes in the Above-Knee Interventions (SAKE) study showed high rate of patency at 6 and 12 months with no stent restenosis for femoropopliteal disease treated with novel interwoven-wire Supera stent [25]. Furthermore, the outcomes after popliteal stenting with the Supera stent from the Leipzing Supera popliteal artery registry showed 6- and 12-month primary patency rate of 94.6% and 87.7%, respectively, with 4 patients experiencing in-stent occlusions and 6 patients experiencing in-stent restenosis [26]. Optimizing stent design can improve stent performance [27].

The length of the SENS is also an important factor of ISR. Long stenosis (> 100 mm) had poor outcome, with restenosis rate exceeding 70% at 1 year [28]. The restenosis rates vary between 35 and 65% at 6–12 months for lesions > 100 cm [29]. In our study, the restenosis rate was significantly higher in the distal portion than in the proximal portion of the long stent (> 100 mm). Conversely, there was no difference in short lesions (< 100 mm). These results revealed the importance of COF caused by oversized SENS in the distal SFA.

Stents are designed to be larger in diameter than the healthy artery, which is referred as “stent oversizing” [19]. The significant diameter change usually occurs in carotid artery stenting, but also occurs in femoral arteries [19]. The mechanical environment of peripheral arteries could be the predominant cause of relatively higher restenosis rate [13]. Although the ideal oversizing ratio is stent specific, nitinol stent oversizing has a very small impact on the immediate lumen gain [30]. However, severe oversizing (stent-to-artery ratio > 1.4:1) resulted in profound long-term histological changes including exuberant neointimal proliferation and luminal stenosis in animal models [6]. These results suggest that the oversizing ratio is an important factor for neointimal formation in SENS, and self-expanding stents without exceeding an oversizing ratio of 1.4 is crucial for long-term patency in peripheral artery stenting [16]. Furthermore, when a non-tapered stent is placed in vessels with a large discrepancy in diameter, attention must be paid for increased neointimal hyperplasia in the oversized side [17]. In our porcine model, the cutoff ratio of SENS to artery ratio was calculated to be 1.51. The restenosis rate was significantly higher in oversized group than in control group.

In summary, we investigated the impact of oversized SENS implanted in tapered SFA arteries on restenosis caused by COF. Oversizing of the stent is an important determinant of final arterial wall diameter and stress. Therefore, SENS with optimal oversizing should be designed in cases with significant arterial tapering.

## Study limitations

In clinical study, our single-center retrospective study design only allowed for analysis based on our routine clinical practice. The sample size did not provide sufficient power to detect ISR incidence or compare clinical events. Operators were not blinded to baseline measures. Finally, our data reflected in-hospital outcomes, and we did not analyze long-term outcomes. Therefore, future randomized studies are needed to confirm our conclusions.

In animal study, the sample size of 22 stents was small, and we did not find significant differences in incidence of restenosis or related parameters between the two groups. In addition, we used healthy animals with non-atherosclerotic arteries. Although blood vessels of pig are very similar to those of human, the results of animal experiments may not be same as those of clinical studies.

## Conclusions

COF is an important cause of restenosis in the distal portion of the SFA stent. Optimal sizing of the SFA stent implantation with SENS is important to reduce the incidence of restenosis. Therefore, COF was an important factor of restenosis following distal SFA stenting.

## Data Availability

The datasets supporting the conclusions of this article are included within the article.
